# Super-localized orthogonal decomposition for convection-dominated diffusion problems

**DOI:** 10.1007/s10543-024-01035-8

**Published:** 2024-08-05

**Authors:** Francesca Bonizzoni, Philip Freese, Daniel Peterseim

**Affiliations:** 1 MOX-Dipartimento di Matematica, Politecnico di Milano, Piazza Leonardo da Vinci 32, 20133 Milan, Italy; 2 Institute of Mathematics, Hamburg University of Technology, Am Schwarzenberg-Campus 3, 21073 Hamburg, Germany; 3 Institute of Mathematics & Centre for Advanced Analytics and Predictive Sciences (CAAPS), University of Augsburg, Universitätsstr. 12a, 86159 Augsburg, Germany

**Keywords:** Convection-dominated diffusion, Numerical homogenization, Multi-scale method, Super-localization, Singularly perturbed, 65N12, 65N15, 65N30, 35B25

## Abstract

This paper presents a novel multi-scale method for convection-dominated diffusion problems in the regime of large Péclet numbers. The method involves applying the solution operator to piecewise constant right-hand sides on an arbitrary coarse mesh, which defines a finite-dimensional coarse ansatz space with favorable approximation properties. For some relevant error measures, including the $L^2$-norm, the Galerkin projection onto this generalized finite element space even yields $\varepsilon$-independent error bounds, $\varepsilon$ being the singular perturbation parameter. By constructing an approximate local basis, the approach becomes a novel multi-scale method in the spirit of the Super-Localized Orthogonal Decomposition (SLOD). The error caused by basis localization can be estimated in an a posteriori way. In contrast to existing multi-scale methods, numerical experiments indicate $\varepsilon$-robust convergence without pre-asymptotic effects even in the under-resolved regime of large mesh Péclet numbers.

## Introduction

This paper studies the numerical solution of the following singularly perturbed convection-diffusion problem in a bounded, simply connected polygonal domain $\varOmega \subset {\mathbb {R}}^d$ with dimension $d=1,2,3$. Given some small diffusivity $0<\varepsilon \ll 1$, a bounded, divergence-free and curl-free velocity field *b* as well as an external force *f*, we look for *u* such that the boundary value problem

1.1 $$\begin{aligned} \left\{ \begin{array}{rl} -\varepsilon \varDelta u+b\cdot \nabla u=f&{}\text {in }\varOmega \\ u=0&{}\text {on }\partial \varOmega \end{array}\right. \end{aligned}$$

holds in suitably weak sense.

This fairly simple model problem appears to be very challenging for classical Galerkin finite element methods (FEMs) and related schemes when the ratio of the convection rate over the diffusion is large, that is, for a large Péclet number $\mathrm{{Pe}}=\left\| b\right\| _{L^\infty (\varOmega )}\varepsilon ^{-1}$. In this regime, the solution *u* typically develops exponential and parabolic layers at the boundary (and possibly interior layers in the presence of inhomogeneous Dirichlet data). Unless the width *h* of the FE mesh resolves the characteristic length scale $1/\mathrm{{Pe}}\approx \varepsilon$ of these layers, FE approximations show spurious oscillations. To avoid this unstable pre-asymptotic behavior, a minimal resolution condition of the form $h\mathrm{{Pe}}\lesssim 1$ is typically required. However, in many relevant practical applications, $\varepsilon$ may be so small that such conditions are unfeasible.

The circumvention or at least relaxation of this resolution condition has been the subject of intensive research in the past few decades. We refer to the monograph [62] for a detailed overview of the subject. Several branches of solution strategies have been developed. One is based on mesh refinement or grading toward layers [3, 31, 54, 55]. The more popular alternative, in particular in engineering communities, is the class of stabilized methods. Roughly speaking, these approaches change the model on the continuum or discrete level by adding artificial diffusion along the negative velocity field (upwinding). Among the extensive number of existing approaches in this context, we mention the streamline upwind/Petrov–Galerkin method [19] (also known as streamline diffusion method - see, e.g., [45]), the Galerkin least-squares method [41], the Douglas–Wang Galerkin method [34], algebraic flux correction [5, 48], discontinuous Petrov–Galerkin methods [26, 51], hybridizable discontinuous Galerkin methods [61], residual-free bubble methods [18, 21], nonconforming stabilized virtual element methods [7] and edge-based methods with additional nonlinear diffusion [4].

It has been observed that many of these stabilized schemes are strongly related to multi-scale methods, marking a third class of approaches to tackle strong convection [43]. The essential idea of multi-scale methods is to resolve the fine-scale features such as strong gradients in the layers by locally precomputed generalized FE shape functions. Prime examples are variational multi-scale methods (VMS) [42, 44, 49, 56], multi-scale FEMs [25, 58], multi-scale hybrid-mixed methods [38], multi-scale discontinuous Galerkin methods [23, 46], multi-scale virtual element methods [64], multi-scale stabilization methods [20, 22], stabilization procedures by means of sub-grid scale [24], energy minimizing generalized multi-scale methods [65], or the multi-scale method for time-dependent convection-dominated problems recently proposed in [63].

Although many of the approaches mentioned so far have been empirically successful in applications and certainly improved on the stability of standard FEMs, $\varepsilon$-independent behaviour is hardly observed for large mesh Péclet numbers $h\mathrm{{Pe}}\gg 1$. This statement also applies to the Localized Orthogonal Decomposition (LOD) method which originated from VMS and is often referred to as numerical homogenization (for an overview on the topic, see [2, 53, 57]). On an ideal level, the methodology realizes a prescribed projection of the unknown solution onto a discrete space (other than the Ritz projection) and hence allows best approximation results in suitable norms independent of the Péclet number. However, existing practical versions based on the localization of the fine-scale Green’s function [27, 43, 60] do suffer from strong convection. Although for moderate mesh Péclet numbers exponential decay results of [47, 52] for the fine-scale Green’s function still apply, they deteriorate with increasing mesh Péclet numbers as outlined in [50]. This prevents the construction of a localized basis by means of fine-scale correctors and limits the practical relevance of the approach.

An alternative localization strategy was recently proposed in [40] for the pure diffusion problem and then extended to indefinite and non-Hermitian problems in [36]. As described in [2], the LOD (and also the VMS) implicitly computes its problem-adapted ansatz space by applying the solution operator to some classical FE spaces on coarse meshes. For the specific choice of piecewise constants, the coarse space is simply given by the span of functions ${\mathscr {A}}^{-1}{\textbf{1}}_T$, ${\mathscr {A}}^{-1}$ denoting the solution operator and ${\textbf{1}}_T$ being the characteristic function of the element *T* ranging into a coarse mesh ${\mathscr {T}}_H$. We refer to the Galerkin projection method on such ansatz space as ideal method. The novel localization strategy aims to identify local linear combinations of characteristic functions in such a way that the spread of the response under the solution operator is minimized. Since for the diffusion model problem this strategy yields a super-exponentially decaying localization error (as compared to the exponentially decaying localization error in classical LOD) the resulting practical method is referred to as Super-Localized Orthogonal Decomposition (SLOD).

The present paper shows that the super-localization strategy is not merely an amplification of the fine-scale Green’s function, but allows localization in applications where it has not been observed before. We generalize the SLOD methodology to convection-diffusion problems with large Péclet number. The SLOD approximation error comprises two contributions: the discretization error of the ideal method and the localization error. As such, the error analysis consists of two major steps. The key result to bound the first contribution is contained in Lemma 2.1, where a priori estimates for the continuous convection-diffusion problem with divergence-free and curl-free velocity field are proved. Thanks to this result, $\varepsilon$-explicit (and in particular cases, even $\varepsilon$-independent) error upper bounds for the ideal method are derived. The second contribution, instead, is proved to be proportional to the computable quantity $\sigma$ (5.5), which reflects the worst-case localization error.

Notably, the SLOD basis functions display an $\varepsilon$-robust behaviour in numerical experiments. Indeed, as $\varepsilon$ gets smaller, they seem not to be affected by oscillations nor an increase of their support seems necessary (see Figs. 2 and 3 for a representation in the one- and two-dimensional frameworks). This represents a major improvement with respect to both classical LOD and the state-of-the-art multi-scale method in [50]. From a practical point of view, this translates into significant computational savings, which in turn makes computations possible even in the three-dimensional framework (see Sect. 7.2 for 3D numerical experiments).

The remainder of the paper is organized as follows. In Sect. 2 a detailed description of the problem of interest in its variational formulation is shown, and a priori upper bounds for the continuous solution of the convection-diffusion problem with divergence-free and curl-free velocity field are proven. An ideal numerical homogenization method based on the $L^2$-orthogonal projection onto piecewise constants is introduced in Sect. 3. The core of the paper are Sects. 4 and 5, where the novel localization approach is presented, turned into a practically feasible method and the error analysis is carried out. Section 6 explains the SLOD algorithm and in Sect. 7 its performances are displayed by means of several two- and three-dimensional numerical experiments.

## Model problem

Let $\varOmega \subset {\mathbb {R}}^d$ be a polygonal, simply connected domain with $d=1,2,3$ and let $0<\varepsilon \le 1$ be a singular perturbation parameter. Moreover, let $b\in L^\infty (\varOmega ;{\mathbb {R}}^d)$ satisfy ${\text {div}}b=0$ and ${\text {curl}}b = 0$. Let $V{:}{=} H^1_0(\varOmega )$ and define the bilinear form $a:V\times V\rightarrow {\mathbb {R}}$ by

2.1 $$\begin{aligned} a(u,v){:}{=} \varepsilon \int _\varOmega \nabla u\cdot \nabla v\, \, \textrm{d}x+ \int _\varOmega (b\cdot \nabla u) v\, \, \textrm{d}x \end{aligned}$$

for all $u,\, v\in V$. Given some linear functional $F\in V'{:}{=} H^{-1}(\varOmega )$ on *V* then the weak formulation of the boundary value problem (1.1) seeks $u\in V$ such that, for all $v\in V$,

2.2 $$\begin{aligned} a(u,v)=F(v). \end{aligned}$$

From now on, we assume that the right-hand side is a bit more regular than minimal, i.e., it is of the form $F(\bullet ){:}{=}\left( f,\bullet \right) _{L^2(\varOmega )}$ for some $f\in L^2(\varOmega )$. This additional regularity of the right-hand side will give rise to orders of approximations. We focus on the convection-dominated regime, namely, $\varepsilon \ll 1$ and Péclet number $\mathrm{{Pe}}=\left\| b\right\| _{L^\infty (\varOmega )}\varepsilon ^{-1}\gg 1$.

### Remark 2.1

The method proposed below naturally applies to the case of non-constant diffusion coefficients, which may incorporate multi-scale features, i.e., the constant diffusivity $\varepsilon$ can be replaced by a variable one of the form $\varepsilon A$ where $A\in L^\infty (\varOmega ;{\mathbb {R}}^{d\times d})$ is symmetric and positive definite almost everywhere in $\varOmega$. Moreover, the method can be generalized to the case of convection-diffusion–reaction equations in a straight-forward way.

Since ${\text {div}}b=0$, integration by parts implies, for all $v\in V$,

2.3 $$\begin{aligned} a(v,v)=\varepsilon \int _\varOmega \left| \nabla v\right| ^2\, \, \textrm{d}x + \int _\varOmega (b\cdot \nabla v) v\, \, \textrm{d}x = \varepsilon \left| v\right| _{V}^2, \end{aligned}$$

where $\left| \bullet \right| _{V}=\left\| \nabla \bullet \right\| _{L^2(\varOmega )}$ denotes the $H^1$-seminorm, which is a norm in *V*. Moreover, for all $u,v\in V$, the application of Cauchy–Schwarz’s and Poincaré’s inequalities readily implies

2.4 $$\begin{aligned} a(u,v)&\le C_a\left| u\right| _{V}\left| v\right| _{V}, \end{aligned}$$

for $C_a=C_a(\varOmega ,\left\| b\right\| _{L^\infty (\varOmega )})=\varepsilon +C_P \left\| b\right\| _{L^\infty (\varOmega )} >0$, where $C_P$ denotes the Poincaré constant. The Lax-Milgram theorem, the coercivity (2.3) and the boundedness (2.4) show that Problem (2.2) admits a unique solution $u\in V$ that satisfies the $\varepsilon$-dependent stability estimate

2.5 $$\begin{aligned} \left| u\right| _{V}\le \frac{C_a}{\varepsilon }\left\| F\right\| _{H^{-1}(\varOmega )}. \end{aligned}$$

For $F(\bullet )=(f,\bullet )_{L^2(\varOmega )}$ and special velocities, the estimate can be sharpened. More importantly, in the weaker $L^2(\varOmega )$-norm, even $\varepsilon$-independent stability results are possible. We refer to [28, Lemma 2.1] which covers the special case $$b = \begin{pmatrix}1&0 \end{pmatrix}^{\top }$$. The subsequent lemma generalizes [28, Lemma 2.1] to velocity fields fulfilling the following technical assumption:

### Assumption 1

The velocity field *b* is both curl- and divergence-free. Moreover, there exists $b_{\min }>0$ such that $|b|\ge b_{\min }$ almost everywhere.

Assumption 1 seems rather restrictive but just extends the assumption on *b* such that we can find a potential field $\psi$ with $b = \nabla \psi$. We introduce $C_\psi = \exp {\left\| \psi \right\| _{L^\infty (\varOmega )}}$. This is crucial for Lemma 2.1 below. Often rigorous numerical analysis requires constant fields or implicitly assume that a result like in Lemma 2.1 below holds. In the numerical experiments, we show that our scheme also applies in the case of more general velocity fields *b*.

Our results are phrased in the $\varepsilon$-scaled norm of *V*

2.6 $$\begin{aligned} \left\| \bullet \right\| _{V,\varepsilon }^2{:}{=}\varepsilon \left| \bullet \right| _{V}^2+\left\| \bullet \right\| _{L^2(\varOmega )}^2, \end{aligned}$$

which is equivalent to the $\left| \bullet \right| _{V}$-norm for $\varepsilon \le 1$, since for all $v\in V$ there holds

2.7 $$\begin{aligned} \sqrt{\varepsilon }\left| v\right| _{V}\le \left\| v\right\| _{V,\varepsilon }\le \sqrt{1+C_P^2}\left| v\right| _{V}. \end{aligned}$$

### Lemma 2.1

Let *b* satisfy Assumption 1, then the unique solution of (2.2) with $f\in L^2(\varOmega )$ satisfies the estimate:

$$\begin{aligned} \left\| u\right\| _{V,\varepsilon } \le \frac{C_{\psi } \sqrt{(1-\varepsilon ) b_{\min }^2 + C_{\psi }^2}}{(1-\varepsilon ) b_{\min }^2} \left\| f\right\| _{L^2(\varOmega )}. \end{aligned}$$

In particular, for $\varepsilon \le \tfrac{1}{2}$, there holds

$$\begin{aligned} \left\| u\right\| _{V,\varepsilon } \le C_{stab} \left\| f\right\| _{L^2(\varOmega )}, \end{aligned}$$

with $C_{stab}$ positive and $\varepsilon$-independent.

### Proof

Following Assumption 1 and using that $\varOmega$ is simply connected, we deduce that $b = \nabla \psi$ for some harmonic potential $\psi$. Now, consider the transformed dependent variable $v(x) = \exp (-\psi (x)) u(x)$, for all $x\in \varOmega$. To derive the strong formulation for *v*, we substitute $u(x) = v(x) \exp (\psi (x))$ in (1.1). This yields:

$$\begin{aligned} -\varepsilon \exp (\psi ) \left( \varDelta v + 2 \nabla \psi \cdot \nabla v + v \left( \left| \nabla \psi \right| _{}^2 + \varDelta \psi \right) \right) + \exp (\psi ) b\cdot \left( \nabla v + v \nabla \psi \right) = f. \end{aligned}$$

As the velocity field is curl- and divergence-free, we get $\nabla \psi = b$ and $\varDelta \psi = 0$. Hence, after multiplying with $v \exp (-\psi )$ and using integration by parts as well as exploiting ${\text {div}}b = 0$, we find

$$\begin{aligned} \varepsilon \left| v\right| _{V}^2 + \left( (1-\varepsilon ) \left| b\right| _{}^2 v,v\right) _{L^2(\varOmega )} = \left( f,\exp (-\psi )v\right) _{L^2(\varOmega )}. \end{aligned}$$

Consequently, we get the following estimate

$$\begin{aligned} \varepsilon \left| v\right| _{V}^2 + (1-\varepsilon ) b_{\textrm{min}}^2 \left\| v\right\| _{L^2(\varOmega )}^2 \le C_{\psi } \left\| f\right\| _{L^2(\varOmega )} \left\| v\right\| _{L^2(\varOmega )} \end{aligned}$$

with the constant $C_{\psi } = \exp {\left\| \psi \right\| _{L^\infty (\varOmega )}}$. This yields a bound on the $L^2(\varOmega )$-norm of *v* as

$$\begin{aligned} \left\| v\right\| _{L^2(\varOmega )} \le \frac{C_{\psi }}{(1-\varepsilon ) b_{\min }^2} \left\| f\right\| _{L^2(\varOmega )}. \end{aligned}$$

Eventually, to bound the $L^2(\varOmega )$-norm of the solution *u*, we use

$$\begin{aligned} \left\| u\right\| _{L^2(\varOmega )} = \left\| \exp (\psi ) v\right\| _{L^2(\varOmega )} \le C_{\psi } \left\| v\right\| _{L^2(\varOmega )} \le \frac{C_{\psi }^2}{(1-\varepsilon ) b_{\min }^2} \left\| f\right\| _{L^2(\varOmega )} \end{aligned}$$

The estimate on the $\left| \bullet \right| _{V}$-norm of the original solution *u* follows by

$$\begin{aligned} \varepsilon \left| u\right| _{V}^2 = a(u,u) = \left( f,u\right) _{L^2(\varOmega )}&\le \left\| f\right\| _{L^2(\varOmega )} \left\| u\right\| _{L^2(\varOmega )} \le C_{\psi } \left\| f\right\| _{L^2(\varOmega )} \left\| v\right\| _{L^2(\varOmega )} \\&\le \frac{C_{\psi }^2}{(1-\varepsilon ) b_{\min }^2} \left\| f\right\| _{L^2(\varOmega )}^2 \end{aligned}$$

By combining the upper bounds on $\varepsilon \left| u\right| _{V}^2$ and $\left\| u\right\| _{L^2(\varOmega )}$, we derive the desired estimate. The $\varepsilon$-independent bound follows directly using $0<\varepsilon \le \frac{1}{2}$ with $C_{stab} = 2 b_{\min }^{-2}C_{\psi } \sqrt{b_{\min }^2 + C_{\psi }^2}$.

$$\square $$

Let us emphasize that the choice of the norm in Lemma 2.1 may be unusual or too weak in the context of convection dominated diffusion problems. However, we are not aware of any global estimates using stronger norms that include the directional derivative. Estimates using norms restricted to subdomains that avoid the layers may be possible, but are the subject of future research.

### Remark 2.2

For the case of a convection-diffusion–reaction equation, the result from Lemma 2.1 is well known, but relies on the presence of the reaction term, see [62, Lemma 1.18]. In this case, as well as the special convection-diffusion case with $$b = \begin{pmatrix} 1&0 \end{pmatrix}^\top $$ also (local) estimates on the directional derivative away from boundary layers are known, see [28, Lemma 1.2] and [62, Remark 1.19].

## An ideal multi-scale method

This section introduces an ideal multi-scale method that identifies an approximation of the solution *u* in an operator-adapted ansatz space $V_H$, whose construction is based on some (possibly coarse) FE mesh.

Let ${\mathscr {T}}_H$ be a (triangular or quadrilateral) shape-regular quasi-uniform mesh (without hanging nodes) of the domain $\varOmega$, where *H* denotes the global mesh size of ${\mathscr {T}}_H$, namely, $H=\max _{T\in {\mathscr {T}}_H} {\text {diam}}(T)$. The degrees of freedom of the multi-scale method are associated with the mesh elements $T\in {\mathscr {T}}_H$ via the characteristic functions ${\textbf{1}}_T$. Given the solution operator ${\mathscr {A}}^{-1}:L^2(\varOmega )\rightarrow V$ that maps each right-hand-side function $f\in L^2(\varOmega )$ to the corresponding unique weak solution of problem (1.1) and the standard FE space

$$\begin{aligned} {\mathbb {P}}^0({\mathscr {T}}_H):= \textrm{span}\left\{ {\textbf{1}}_T\, |\, T\in {\mathscr {T}}_H\right\} \end{aligned}$$

of ${\mathscr {T}}_H$-piecewise constants, the finite-dimensional subspace $V_H\subset V$ is given by

3.1 $$\begin{aligned} V_H:={\mathscr {A}}^{-1}{\mathbb {P}}^0({\mathscr {T}}_H)=\textrm{span}\left\{ {\mathscr {A}}^{-1}{\textbf{1}}_T\, |\, T\in {\mathscr {T}}_H\right\} . \end{aligned}$$

Note that we could have chosen FE spaces other than ${\mathbb {P}}^0({\mathscr {T}}_H)$ for the approximation of the right-hand side. For example, the paper [27] considers discontinuous piecewise linears on simplicial meshes and [56] considers continuous piecewise linears with zero boundary condition. More generally, a finite-dimensional space of linear functionals on *V* could be considered. The authors in [50] implicitly use the Dirac delta functions $\delta _z$ for the interior vertices *z* of ${\mathscr {T}}_H$. Clearly this is only possible in one dimension and requires regularization in higher dimensions. While in two dimensions this was somewhat justifiable, the three-dimensional case seemed not to be tractable with this choice.

Let $\Pi _H:L^2(\varOmega )\rightarrow {\mathbb {P}}^0({\mathscr {T}}_H)$ denote the $L^2$-orthogonal projection operator and note that, for all $T\in {\mathscr {T}}_H$, $\Pi _H v|_T$ is given by

$$\begin{aligned} \Pi _H v|_T=\frac{1}{|T|}\int _T v\, \, \textrm{d}x. \end{aligned}$$

It is well-known that $\Pi _H$ fulfills the following local stability and approximation properties (see [6, 59])

3.2 $$\begin{aligned} \left\| \Pi _H v\right\| _{L^2(T)}&\le \left\| v\right\| _{L^2(T)}\quad \text {for all }\, v\in L^2(T), \end{aligned}$$

3.3 $$\begin{aligned} \left\| v-\Pi _H v\right\| _{L^2(T)}&\le \pi ^{-1} H \left\| \nabla v\right\| _{L^2(T)}\quad \text {for all }\, v\in H^1(T). \end{aligned}$$

Given the kernel ${\mathscr {W}}{:}{=}\ker (\Pi _H\vert _V)$ of $\Pi _H$ when restricted to *V*, we may identify the decomposition

3.4 $$\begin{aligned} V= V_H \oplus {\mathscr {W}},\quad a(V_H,{\mathscr {W}}) = 0, \end{aligned}$$

which justifies the term orthogonal decomposition in the name of the method.

We will not use this decomposition explicitly in this paper, but our derivation is based on the composition of the $L^2$ orthogonal projection $\Pi _H$ and the solution operator ${\mathscr {A}}^{-1}$, which defines an ideal multi-scale method that maps right-hand sides $f\in L^2(\varOmega )$ into $V_H$. Thus, $u_H={\mathscr {A}}^{-1}\Pi _H f$ satisfies $a(u_H,v)=\left( \Pi _H f,v\right) _{L^2(\varOmega )}$ for all $v\in V$. Since $u_{H}$ is in $V_{H}$ by construction, it coincides with its Galerkin projection onto $V_{H}$, i.e., $u_H\in V_H$ is equivalently and uniquely characterized by the discrete variational problem

3.5 $$\begin{aligned} a(u_H,v_H)=\left( \Pi _H f,v_H\right) _{L^2(\varOmega )} \end{aligned}$$

for all $v_H\in V_H$. Note that this is a non-standard projection of the solution *u* onto the discrete space $V_H$. The method differs slightly from the classical Galerkin LOD or its Petrov–Galerkin variants. It equals the Galerkin projection and the abstract Petrov–Galerkin framework of [2] only for $f\in {\mathbb {P}}^0({\mathscr {T}}_H)$. For general $f\in L^2(\varOmega )$ it differs from the more established variants. In the pure diffusion case it equals the collocation variant discussed in [40].

In the following lemma we derive an $\varepsilon$-independent upper bound on the discretization error under Assumption 1, which is the motivation for the particular choice of the LOD variant (3.5).

### Lemma 3.1

Let $f\in H^s(\varOmega )$ with $s\in [0,1]$, and *b* as in Assumption 1. Also assume that the assumption on $\varepsilon$ from Lemma 2.1 is satisfied. Denote with $u\in V$ and $u_H\in V_H$ the unique solutions to (2.2) and (3.5), respectively. Then, there holds

3.6 $$\begin{aligned} \left\| u-u_H\right\| _{V,\varepsilon } \le C_{stab}\left\| f-\Pi _H f\right\| _{L^2(\varOmega )} \le C\, C_{stab}H^s\left\| f\right\| _{H^s(\varOmega )}, \end{aligned}$$

where *C*, $C_{stab}$ are $\varepsilon$- and *H*-independent positive constants, $C_{stab}$ being introduced in Lemma 2.1.

### Proof

Since $u={\mathscr {A}}^{-1}f$ and $u_H={\mathscr {A}}^{-1}\Pi _H f$ we readily get

$$\begin{aligned} \left\| u-u_H\right\| _{V,\varepsilon }&=\left\| {\mathscr {A}}^{-1}f-{\mathscr {A}}^{-1}\Pi _H f\right\| _{V,\varepsilon } =\left\| {\mathscr {A}}^{-1}(f-\Pi _H f)\right\| _{V,\varepsilon }. \end{aligned}$$

Lemma 2.1 provides an upper bound of the right-hand side. Altogether,

$$\begin{aligned} \left\| u-u_H\right\| _{V,\varepsilon } \le C_{stab}\left\| f-\Pi _H f\right\| _{L^2(\varOmega )} \le C\,C_{stab}H^s\left\| f\right\| _{H^s(\varOmega )}, \end{aligned}$$

where the last inequality holds for all right-hand sides $f\in H^s(\varOmega )$ with $s\in [0,1]$. $\square$

Apart the exactness of the ideal method for $f\in {\mathbb {P}}^0({\mathscr {T}}_H)$, Lemma 3.1 above contains an error bound in the weaker $L^2(\varOmega )$-norm that is independent of $\varepsilon$. First order convergence is predicted without a pre-asymptotic regime. The numerical experiments of the later sections will rather report second order and even $\varepsilon$-independent first order for the $H^1(\varOmega )$-seminorm. A more abstract version of the estimate of (3.6) reads

$$\begin{aligned} \left\| u-u_H\right\| _{Y} \le \Vert {\mathscr {A}}^{-1}\Vert _{X\rightarrow Y}\left\| f-\Pi _H f\right\| _{X}, \end{aligned}$$

where $\Vert {\mathscr {A}}^{-1}\Vert _{X\rightarrow Y}$ refers to the norm of ${\mathscr {A}}^{-1}$ as a mapping between suitable Banach spaces *X* and *Y*. Choosing $X=H^{-1}(\varOmega )$ and $Y=L^2(\varOmega )$ or $Y=H^1(\varOmega )$ or $Y=H^1(\omega )$ where $\omega \subset \varOmega$ excludes the boundary layers would pave the way to proving the numerically observed rates. However, we are not aware of any $\varepsilon$-independent bounds of the required operator norms.

## Super-localization of basis functions

The canonical basis functions $\{{\mathscr {A}}^{-1}{\textbf{1}}_T\,|\, T\in {\mathscr {T}}_H\}$ of the operator-adapted approximation space $V_H$ are non-local. To make the method practically feasible, localized basis functions have to be identified. The LOD provides a mechanism to construct an exponentially decaying basis that has been very successful in many applications. However, this is not the case when applied to convection-dominated problems, as we are interested here. More precisely, when applying the abstract theory of [2] the exponential decay property deteriorates as $\varepsilon$ goes to 0, and the error estimate of error committed by computing a localized approximation of the exponentially decaying basis is only shown to behave like $\varepsilon ^{-1}H^{-1-d/2}\exp (-c\varepsilon \ell )$. This indicates that the localization parameter needs to grow algebraically in $\varepsilon ^{-1}$ to make this quantity small. This is in line with practical experience, documented, e.g., in [50]. Therein, the authors also discuss a possible improvement using anisotropic patches. However, the construction is based on point evaluation functionals and, hence, essentially limited to the one- and two-dimensional case.

This section presents an advanced localization strategy, which has superior properties, yielding, in particular, super-exponential decay of the localization error. The main idea stays in the identification of local ${\mathscr {T}}_H$-piecewise constant source terms that yield rapidly decaying (or even local) responses under the solution operator ${\mathscr {A}}^{-1}$ of the convection-dominated problem (1.1). This super-localization strategy, now known as the Super-Localized Orthogonal Decomposition (SLOD), has been first introduced in [40] for the second order elliptic partial differential equation $-{\text {div}}(A\nabla u)=f$, and subsequently extended to indefinite non-hermitian problems in [36].

For the subsequent derivation of the super-localization strategy, we need to introduce some notations. The local patch of level $\ell \in {\mathbb {N}}$ of a union of elements $S\subset \varOmega$ is given by:

$$\begin{aligned} N^\ell (S){:}{=} {\left\{ \begin{array}{ll} \bigcup \{T\in {\mathscr {T}}_H\,|\, T\cap S\ne \emptyset \} &{} \ell =1\\ N^1(N^{\ell -1}(S)) &{} \ell =2,3,4,\ldots \end{array}\right. } \end{aligned}$$

Let $\ell \in {\mathbb {N}}$ be fixed, such that no patch coincides with the entire domain $\varOmega$. Given $T\in {\mathscr {T}}_H$, denote

- $\omega {:}{=} N^\ell (T)$ its $\ell$-th order patch;
- $V_\omega {:}{=} \left\{ v|_\omega \hspace{1ex}\vert \, v\in V \right\}$ the restriction of *V* to the patch $\omega$, equipped with the semi-norm $\left| \bullet \right| _{H^1(\omega )}$ and the norm $\left\| \bullet \right\| _{H^1(\omega )}$;
- ${\mathscr {T}}_{H,\omega }{:}{=} \{K\in {\mathscr {T}}_H\cap \omega \}$ the sub-mesh of ${\mathscr {T}}_H$ with elements in $\omega$;
- $\Pi _{H,\omega }:L^2(\varOmega )\rightarrow {\mathbb {P}}^0({\mathscr {T}}_{H,\omega })$ the $L^2$-orthogonal projection onto ${\mathbb {P}}^0({\mathscr {T}}_{H,\omega })$.

Note that throughout the paper we will not distinguish between the functions in $H^1_0(\omega )$ and their *V*-conforming extension by 0 to the entire domain $\varOmega$.

The (ideal) basis function $\varphi =\varphi _{T,\ell ,\varepsilon }\in V_H$ associated with the element *T* is given by the ansatz

$$\begin{aligned} \varphi ={\mathscr {A}}^{-1} g\quad \textrm{with}\quad g=g_{T,\ell ,\varepsilon }{:}{=} \sum _{K\in {\mathscr {T}}_{H,\omega }} c_K{\textbf{1}}_K, \end{aligned}$$

for some coefficients $(c_K)_{K\in {\mathscr {T}}_{H,\omega }}$ that will be determined later. In particular, $\varphi$ fulfils, for all $v\in V$,

$$\begin{aligned} a(\varphi ,v)=\left( g,v\right) _{L^2(\omega )}. \end{aligned}$$

The Galerkin projection of $\varphi$ onto the local subspace $H^1_0(\omega )$ is the function $\varphi ^\textrm{loc}=\varphi ^\textrm{loc}_{T,\ell ,\varepsilon }\in H^1_0(\omega )$ that satisfies, for all $v\in H^1_0(\omega )$,

4.1 $$\begin{aligned} a_\omega (\varphi ^\textrm{loc},v)=\left( g,v\right) _{L^2(\omega )}, \end{aligned}$$

where $a_\omega (\cdot ,\cdot )$ denotes the restriction of the bilinear form $a(\cdot ,\cdot )$ to the subset $\omega$. In general, the local function $\varphi ^\textrm{loc}$ is a poor approximation of the ideal function $\varphi$. Nevertheless, appropriate nontrivial choices of *g* (i.e., of coefficients $(c_K)_{K\in {\mathscr {T}}_{H,\omega }}$) lead to highly accurate approximations in the energy norm.

The quantity we aim to minimize is the localization error $\varphi - \varphi ^\textrm{loc}$, i.e. the error between the ideal basis function $\varphi$ and its localized counterpart $\varphi ^\textrm{loc}$. Note that the patch $\omega$ is a polytope. Hence, following [30, Theorem 31.31, Theorem 31.33], $\varphi ^\textrm{loc}\in H^{1+s}(\omega )$ for some $s>\tfrac{1}{2}$. The normal derivative is therefore integrable [29, Example 4.16], and we may derive the following lemma.

### Lemma 4.1

(Variational characterization of the localization error) Let *n* be the outward normal of $\omega$. There holds for all $v\in V$

$$\begin{aligned} a(\varphi -\varphi ^\textrm{loc},v) = - \varepsilon \int _{\partial \omega \setminus \partial \varOmega } n \cdot \nabla \varphi ^\textrm{loc} v \, \textrm{d}s. \end{aligned}$$

Moreover, we get a bound for the localization error as:

$$\begin{aligned} \left| \varphi - \varphi ^\textrm{loc}\right| _{V} \le C\sqrt{\frac{C_{P}^2 + 1}{\ell H}} \left\| n \cdot \nabla \varphi ^\textrm{loc}\right\| _{L^2(\partial \omega \setminus \partial \varOmega )}. \end{aligned}$$

### Proof

Let $v\in V$. Then, using the definitions of $\varphi$ and $\varphi ^\textrm{loc}$ and integration by parts, there holds:

$$\begin{aligned} a(\varphi -\varphi ^\textrm{loc},v)&= a(\varphi ,v) - a(\varphi ^\textrm{loc},v) = \left( g,v\right) _{L^2(\omega )} - a_\omega (\varphi ^\textrm{loc},v)\\&= \left( g,v\right) _{L^2(\omega )} - \varepsilon \int _\omega \nabla \varphi ^\textrm{loc}\cdot \nabla v \, \textrm{d}x - \int _\omega (b \cdot \nabla \varphi ^\textrm{loc}) v \, \textrm{d}x \\&= \left( g,v\right) _{L^2(\omega )} - \int _\omega \left( - \varepsilon \varDelta \varphi ^\textrm{loc}+ (b \cdot \nabla \varphi ^\textrm{loc}) \right) v \, \textrm{d}x\\&\quad - \varepsilon \int _{\partial \omega \setminus \partial \varOmega } (n\cdot \nabla \varphi ^\textrm{loc}) v \, \textrm{d}s \\&= - \varepsilon \int _{\partial \omega \setminus \partial \varOmega } (n\cdot \nabla \varphi ^\textrm{loc}) v \, \textrm{d}s . \end{aligned}$$

In particular, for $e^\textrm{loc} = \varphi - \varphi ^\textrm{loc}$, we find

$$\begin{aligned} \varepsilon \left| e^\textrm{loc}\right| _{V}^2&= a(e^\textrm{loc}, e^\textrm{loc})\\&\le \left| \varepsilon \int _{\partial \omega \setminus \partial \varOmega } (n\cdot \nabla \varphi ^\textrm{loc}) e^\textrm{loc} \, \textrm{d}s\right| _{} \le \varepsilon \left\| n \cdot \nabla \varphi ^\textrm{loc}\right\| _{L^2(\partial \omega \setminus \partial \varOmega )} \left\| e^\textrm{loc}\right\| _{L^2(\partial \omega \setminus \partial \varOmega )}. \end{aligned}$$

Now, using the trace inequality (note that in the current setting we consider here, this dependence is of the form $C \sqrt{\ell H}$ with some constant $C>0$ independent of *H* and $\ell$), we get

$$\begin{aligned}&\varepsilon \left| e^\textrm{loc}\right| _{V}^2 \le \frac{\varepsilon C}{\sqrt{\ell H}} \left\| n \cdot \nabla \varphi ^\textrm{loc}\right\| _{L^2(\partial \omega \setminus \partial \varOmega )} \left\| e^\textrm{loc}\right\| _{H^1(\omega )} \\&\quad \le \frac{\varepsilon C}{\sqrt{\ell H}} \left\| n \cdot \nabla \varphi ^\textrm{loc}\right\| _{L^2(\partial \omega \setminus \partial \varOmega )} \left\| e^\textrm{loc}\right\| _{H^1(\varOmega )}. \end{aligned}$$

Finally, from Poincarés inequality we find

$$\begin{aligned} \varepsilon \left| e^\textrm{loc}\right| _{V}^2 \le \varepsilon C \sqrt{\frac{{C_{P}^2+1}}{{\ell H}}} \left\| n \cdot \nabla \varphi ^\textrm{loc}\right\| _{L^2(\partial \omega \setminus \partial \varOmega )} \left| e^\textrm{loc}\right| _{V}, \end{aligned}$$

which yields the final result. $\square$

### Remark 4.1

In previous work [36, 40], the smallness of the normal derivative has been interpreted as the (almost) $L^2$-orthogonality of *g* with respect to the space of convection-harmonic functions. Here, however, we directly use the smallness of the normal derivative, which makes the algorithm even simpler and avoids the sampling of the respective space of convection-harmonic functions.

Lemma 4.1 gives rise to the correct definition of the coefficients $(c_K)_{K\in {\mathscr {T}}_{H,\omega }}$. Namely, we choose $(c_K)_{K\in {\mathscr {T}}_{H,\omega }}$ that minimize $\left\| n \cdot \nabla \varphi ^\textrm{loc}\right\| _{L^2(\partial \omega {\setminus } \partial \varOmega )}$ subject to $\left\| g\right\| _{L^2(\omega )} = 1$. For the vector *c* consisting of the coefficients $c_{K}$, this optimization task can be written in matrix form as follows:

4.2 $$\begin{aligned} \min _{c} c^\top N c\quad \text {subject to } c^\top B c = 1, \end{aligned}$$

where the entries of the matrix *N* are $(N)_{i,j} = \int _{\partial \omega {\setminus } \partial \varOmega } (n\cdot \nabla \varphi ^\textrm{loc}_{i}) (n\cdot \nabla \varphi ^\textrm{loc}_{j}) \, \textrm{d}s$ and $B = H^d I$, *I* being the identity matrix. Equation (4.2) can be equivalently written as the following generalized eigenvalue problem: Find the smallest eigenvalue $\lambda$ and the corresponding eigenvector *c* such that

4.3 $$\begin{aligned} Nc = \lambda Bc. \end{aligned}$$

More details on the precise choice of the basis functions is given in Sect. 6.

**Fig. 1:**
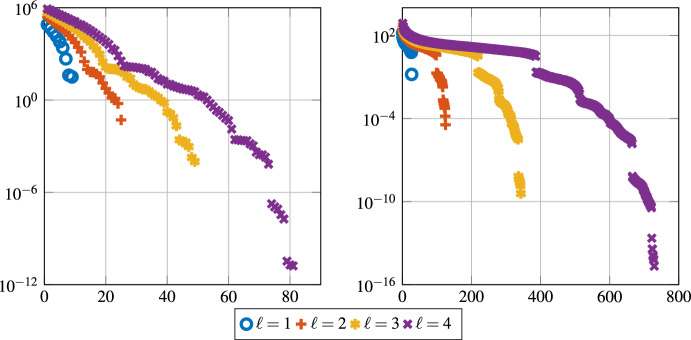
All eigenvalues of the pair of matrices (*N*, *B*) for different values of the localization parameter $\ell$, for a patch that does not reach the boundary . (left) Two-dimensional result for $$ b = \sqrt{\tfrac{2}{5 + \sqrt{5}}} \begin{pmatrix} \tfrac{1+\sqrt{5}}{2}&1 \end{pmatrix}^\top $$ and $\varepsilon = 2^{-11}$ on a coarse mesh $H = 2^{-4}$. (right) Three-dimensional result for $$ b = \sqrt{\tfrac{2}{7 + \sqrt{5}}} \begin{pmatrix} \tfrac{1+\sqrt{5}}{2}&1&1 \end{pmatrix}^\top $$ and $\varepsilon = 2^{-6}$ on a coarse mesh with $H=2^{-4}$

For the case of pure diffusion [40] there is strong numerical evidence that a quantity related to the $L^2(\partial \omega \setminus \partial \varOmega )$-norm of the normal derivative decays super-exponentially in $\ell$. Also in the presence of convection and even for high Péclet numbers, the numerical experiments in Fig. 1 show super-exponential decay of the smallest eigenvalue $\lambda$ solution of problem (4.3), with respect to the localization parameter $\ell$. As a consequence, there is a super-exponential decay of the normal derivative $\left\| n \cdot \nabla \varphi ^\textrm{loc}\right\| _{L^2(\partial \omega {\setminus } \partial \varOmega )}$ of the corresponding localized basis functions in the localization parameter $\ell$. For the limit $\varepsilon \rightarrow 0$, however, we believe that this decay deteriorates, and especially for the pure transport case, such a decay is not expected. However, in the presence of some diffusion in the model, the decay seems to be (super-)exponential. Henceforth, we assume that there exist *g* with $\left\| g\right\| _{L^2(\omega )} = 1$ and constants $C_{sd}(\varepsilon ,H,\ell )>0$ depending on $\varepsilon ,\, H$ and $\ell$, but independent of *T*, and $C>0$ independent of $H,\, \ell$ and *T* such that

4.4 $$\begin{aligned} \left\| {n \cdot \nabla } \varphi ^\textrm{loc}\right\| _{L^2(\partial \omega \setminus \partial \varOmega )} \le C_{sd}(\varepsilon ,H,\ell ) \exp \left( -C(\varepsilon )\ell ^{\frac{d}{d-1}}\right) . \end{aligned}$$

### Remark 4.2

**Fig. 2:**
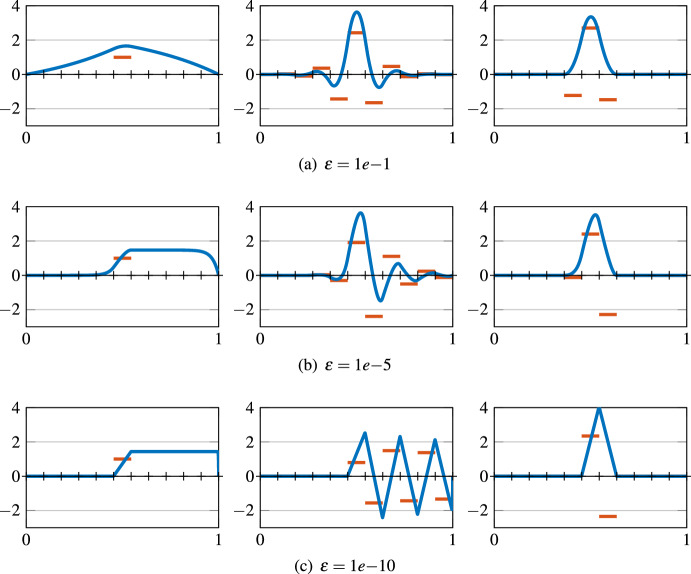
Solution to the convection-dominated problem with right-hand side ${\textbf{1}}_T$, i.e., ${\mathscr {A}}^{-1}{\textbf{1}}_T$ (left); $L^2$-projection based (global) LOD basis function (center); Novel SLOD basis function (right). Their corresponding $L^2$-normalized right-hand sides are depicted in orange

(SLOD basis in 1d) In the one-dimensional case, the boundary of the patches consists only of the two end points of the respective intervals, whereas we have three degrees of freedom for an order $\ell =1$ patch. Thus, the minimization problem (4.2) can be solved exactly, which yields a vanishing normal derivative on both end points of the patches. Hence, from (4.4) for $d=1$, interpreting $\frac{d}{d-1}$ as infinity, reveals a truly local basis function. This effect is also observed in Fig. 2, where we compare three different basis functions in $V_{H}$ for various values of $\varepsilon$ and corresponding to the same mesh element $T\in {\mathscr {T}}_H$, namely ${\mathscr {A}}^{-1}{\textbf{1}}_T$ (left); the basis function for $L^2$-projection based LOD (center); the SLOD basis function $\varphi ^\textrm{loc}_{T,1,\varepsilon }$ (right).

### Remark 4.3

**Fig. 3:**
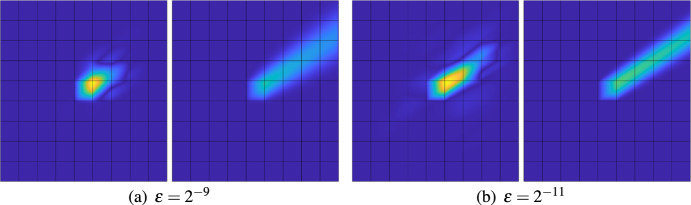
Absolute value of SLOD basis (left) and solution of ${\mathscr {A}}^{-1}{\textbf{1}}_T$ (right) on 4-th order (interior) patch, for $$b = \sqrt{\tfrac{2}{5 + \sqrt{5}}} \begin{pmatrix} \tfrac{1+\sqrt{5}}{2}&1 \end{pmatrix}^\top $$ and different values of $\varepsilon$

(SLOD basis in 2d and 3d) While in the one-dimensional setting we were able to retrieve truly local basis functions, this is no longer true in higher dimensions. In Fig. 3 we depict the basis functions $\varphi ^\textrm{loc}_{T,4,\varepsilon }$ for an element *T* whose patch does not reach the global boundary for $\varepsilon = 2^{-9}$ and $\varepsilon = 2^{-11}$. The velocity field *b* is given as $$b = \sqrt{\tfrac{2}{5 + \sqrt{5}}} \begin{pmatrix} \tfrac{1+\sqrt{5}}{2}&1 \end{pmatrix}^\top $$. Moreover, the figure shows the response of the solution operator to the indicator function ${\textbf{1}}_{T}$ that corresponds to *T*. It is clearly visible, that the SLOD basis functions decay very fast, especially in comparison to the ideal basis functions of the space (3.1).

## Super-localized multi-scale method and error analysis

Within this section we turn the method (3.5) based on the ideal operator-adapted ansatz subspace $V_H\subset V$ into a feasible numerical scheme, by means of the super-localization strategy introduced above.

Let the localization parameter $\ell$ be fixed. We define the ansatz space of the super-localized method as the span of the SLOD basis functions $\varphi _{T,\ell ,\varepsilon }^\textrm{loc}$ as *T* varies in the coarse grid ${\mathscr {T}}_H$, namely:

5.1 $$\begin{aligned} V_{H}^{\ell }{:}{=} \left\{ \varphi _{T,\ell ,\varepsilon }^\textrm{loc}\,|\,T\in {\mathscr {T}}_H\right\} \subset V. \end{aligned}$$

The approximate solution provided by the SLOD method is the Galerkin projection in the space $V_{H}^{\ell }$ of the convection-dominated problem at hand with perturbed right-hand side $\Pi _H f$. In particular, the SLOD approximation to (2.2) is the function $u_{H}^{\ell }\in V_{H}^{\ell }$ such that, for all $v_{H}^{\ell }\in V_{H}^{\ell }$,

5.2 $$\begin{aligned} a(u_{H}^{\ell },v_{H}^{\ell })=\left( \Pi _H f,v_{H}^{\ell }\right) _{L^2(\varOmega )}. \end{aligned}$$

We emphasize that we can expand $\Pi _Hf\in {\mathbb {P}}^0({\mathscr {T}}_H)$ in the basis of right-hand sides $\left\{ g_{T,\ell ,\varepsilon }\,|\,T\in {\mathscr {T}}_H\right\}$, which yields

5.3 $$\begin{aligned} \Pi _Hf=\sum _{T\in {\mathscr {T}}_H}c_T g_{T,\ell ,\varepsilon }. \end{aligned}$$

A minimal requirement for the stability and convergence of the Galerkin method (5.2) is that the set of functions $\left\{ g_{T,\ell ,\varepsilon }\,|\,T\in {\mathscr {T}}_H \right\}$ spans ${\mathbb {P}}^0({\mathscr {T}}_H)$ in a stable way. Numerically, this is ensured as described in Sect. 6. For the subsequent theoretical analysis, we make the following assumption.

### Assumption 2

The set $\left\{ g_{T,\ell ,\varepsilon }\,|\,T\in {\mathscr {T}}_H\right\}$ is a Riesz basis of ${\mathbb {P}}^0({\mathscr {T}}_H)$, i.e., there exists a constant $C_{rb}(\varepsilon ,H,\ell )$, depending only polynomially on *H* and $\ell$, such that, for all $\{c_T\}_{T\in {\mathscr {T}}_H}\subset {\mathbb {R}}$, there holds

5.4 $$\begin{aligned} C_{rb}^{-1}(\varepsilon ,H,\ell )\sum _{T\in {\mathscr {T}}_H}|c_T|^2 \le \left\| \sum _{T\in {\mathscr {T}}_H}c_T g_{T,\ell ,\varepsilon }\right\| _{L^2(\varOmega )}^2 \le C_{rb}(\varepsilon ,H,\ell )\sum _{T\in {\mathscr {T}}_H}|c_T|^2. \nonumber \\ \end{aligned}$$

In the following theorem we derive an a priori error estimate for the solution to problem (5.2). The upper bound is explicit in the quantity

5.5 $$\begin{aligned} \sigma (\varepsilon ,H,\ell ){:}{=} \max _{T\in {\mathscr {T}}_H} \left\| {n\cdot \nabla } \varphi ^\textrm{loc}_{T,\ell ,\varepsilon }\right\| _{L^2(\partial \omega \setminus \partial \varOmega )} \end{aligned}$$

which reflects the worst-case localization error.

### Remark 5.1

(Exponential decay of classical LOD) For moderate mesh Péclet number, the quantity $\sigma (\varepsilon ,H,\ell )$ in (5.5) decays exponentially in the localization parameter $\ell$ (see [40, Appendix A] for the proof in the pure diffusion case). In particular, one can recover the a priori error estimate with rates similar to those for the LOD theory as in [2, 27, 50].

### Theorem 5.1

(Convergence of the SLOD method) Let Assumption 2 and Assumption 1 be satisfied. Then, there exists a constant $C>0$ independent of $H,\,\ell ,\,\varepsilon$ such that, for all $f\in H^s(\varOmega )$ with $s\in [0,1]$, there holds

5.6 $$\begin{aligned}&\left\| u-u_{H}^{\ell }\right\| _{V,\varepsilon } \nonumber \\&\le C\left( C_{stab}\left\| f-\Pi _H f\right\| _{L^2(\varOmega )} + {\frac{\sigma (\varepsilon ,H,\ell )}{\varepsilon \sqrt{\ell H}}} C_{rb}(\varepsilon ,H,\ell )^{1/2}\ell ^{d/2}\left\| f\right\| _{L^2(\varOmega )} \right) \nonumber \\&\le C\left( H^s\left\| f\right\| _{H^s(\varOmega )} + {\frac{\sigma (\varepsilon ,H,\ell )}{\varepsilon \sqrt{\ell H}}} C_{rb}(\varepsilon ,H,\ell )^{1/2}\ell ^{d/2}\left\| f\right\| _{L^2(\varOmega )} \right) , \end{aligned}$$

where $C_{rb}(\varepsilon ,H,\ell )$ and $\sigma (\varepsilon ,H,\ell )$ are defined in Assumption 2 and (5.5), respectively.

### Proof

By triangular inequality, we get:

5.7 $$\begin{aligned} \left\| u-u_{H}^{\ell }\right\| _{V,\varepsilon }\le \left\| u-u_H\right\| _{V,\varepsilon } + \left\| u_H-u_{H}^{\ell }\right\| _{V,\varepsilon }. \end{aligned}$$

The first term in (5.7) represents the discretization error of the ideal multi-scale method, and its upper bound is given by Lemma 3.1. We consider now the second term in (5.7), which represents the localization error. Observe that $u_H$ solves the continuous equation for right-hand side $\Pi _Hf$. As a consequence, the SLOD solution $u_{H}^{\ell }$ is the Galerkin approximation of $u_H$ in the finite dimensional space $V_{H}^{\ell }$. Using the norm equivalence (2.7) and applying Céa’s Lemma, we get

$$\begin{aligned} \left\| u_H-u_{H}^{\ell }\right\| _{V,\varepsilon } \lesssim \left| u_H-u_{H}^{\ell }\right| _{V} \lesssim \frac{1}{\varepsilon } \inf _{v_{H}^{\ell }\in V_{H}^{\ell }}\left| u_H-v_{H}^{\ell }\right| _{V}, \end{aligned}$$

where the notation $x\lesssim y$ means $x\le c y$ with *c* positive constant independent of the mesh size parameter *H*, the localization parameter $\ell$ and the diffusion coefficient $\varepsilon$. Given the expansion of $\Pi _Hf$ in the basis $\left\{ g_{T,\ell ,\varepsilon }\,|\,T\in {\mathscr {T}}_H\right\}$, namely, $\Pi _Hf=\sum _{T\in {\mathscr {T}}_H}c_T g_{T,\ell ,\varepsilon }$, we can express $u_H$ as

$$\begin{aligned} u_H=\sum _{T\in {\mathscr {T}}_H}c_T{\mathscr {A}}^{-1}g_{T,\ell ,\varepsilon } =\sum _{T\in {\mathscr {T}}_H}c_T\varphi _{T,\ell ,\varepsilon }. \end{aligned}$$

For the particular choice $v_{H}^{\ell }=\sum _{T\in {\mathscr {T}}_H}c_T\varphi ^{loc}_{T,\ell ,\varepsilon }$, we obtain that $e{:}{=} u_H-v_{H}^{\ell }\in V$ fulfils:

$$\begin{aligned} \left| e\right| _{V}^2&=\frac{1}{\varepsilon }a(u_H-v_{H}^{\ell },e) =\frac{1}{\varepsilon }\sum _{T\in {\mathscr {T}}_H}c_T a(\varphi _{T,\ell ,\varepsilon }-\varphi _{T,\ell ,\varepsilon }^{loc},e)\\&{= - \sum _{T\in {\mathscr {T}}_H}c_T \int _{\partial \omega } n \cdot \nabla \varphi ^\textrm{loc}_{T,\ell ,\varepsilon } e \, \textrm{d}s} \\&\le \sum _{T\in {\mathscr {T}}_H} \left| c_T\right| \left\| {n\cdot \nabla } \varphi _{T,\ell ,\varepsilon }^\textrm{loc}\right\| _{L^2(\partial \omega )} {\frac{C}{\sqrt{\ell H}}} \left\| e\right\| _{H^1(\omega )}\\&\le \sigma (\varepsilon ,H,\ell ) {\frac{C}{\sqrt{\ell H}}} \sum _{T\in {\mathscr {T}}_H} \left| c_T\right| \left\| e\right\| _{H^1(\omega )}, \end{aligned}$$

where we employed Lemma 4.1 in the third equality and (5.5) in the last inequality. For simplicity, we omit the dependence of $\sigma$ and $C_{rb}$ on $\varepsilon ,H$ and $\ell$ in the rest of the proof. As a consequence, thanks to Assumption 2, (5.3), the Poincaré inequality and (3.2), there holds:

$$\begin{aligned} \left| e\right| _{V}^2&\lesssim \frac{\sigma }{{\sqrt{\ell H}}} \sum _{T\in {\mathscr {T}}_H} \left| c_T\right| \left\| e\right\| _{H^1(\omega )} \lesssim \frac{\sigma }{{\sqrt{\ell H}}} \sqrt{\sum _{T\in {\mathscr {T}}_H} \left| c_T\right| ^2} \sqrt{\sum _{T\in {\mathscr {T}}_H}\left\| e\right\| _{H^1(\omega )}^2}\\&\lesssim \frac{\sigma }{{\sqrt{\ell H}}} \left( C_{rb}^{1/2}\left\| \Pi _Hf\right\| _{L^2(\varOmega )}\right) \left( C_{ol}\ell ^{d/2}\left\| e\right\| _{H^1(\varOmega )}\right) \\&\lesssim \frac{\sigma }{{\sqrt{\ell H}}} \left( C_{rb}^{1/2}\left\| f\right\| _{L^2(\varOmega )}\right) \left( C_{ol}\ell ^{d/2}\right) \left| e\right| _{V}, \end{aligned}$$

where $C_{ol}^2\ell ^d$ bounds the number of patches containing a fixed mesh element. In particular, we have proved that

$$\begin{aligned} \left| e\right| _{V}\lesssim \frac{\sigma }{{\sqrt{\ell H}}} C_{rb}^{1/2} \ell ^{d/2}\left\| f\right\| _{L^2(\varOmega )}, \end{aligned}$$

so that the estimate (5.6) follows. $\square$

As previously observed for the ideal multi-scale method, upper bounds on the SLOD error could be derived in the abstract setting ${\mathscr {A}}^{-1}:X\rightarrow Y$, for suitable Banach spaces *X* and *Y*.

We point out that in the case of a piecewise constant right-hand side *f*, the first term in (5.6) vanishes. From our assumption on the decay of the normal derivative in (4.4), we deduce that the $\varepsilon$-dependence of the second expression is dominated by the exponentially decaying quantity $\sigma (\varepsilon ,H,\ell )$. Moreover, we derive that the localization condition $\ell \gtrsim \left| \log (\varepsilon H)\right| ^{\frac{d-1}{d}}$ guarantees convergence of the SLOD with order *H*. In the limit $\varepsilon \rightarrow 0$, this would still result in a dense matrix.

## Numerical implementation and stable selection of basis

This section discusses the implementation of the proposed numerical method, with particular attention to the computation of a basis $\{\varphi _{T,\ell ,\varepsilon }^\textrm{loc}\,|\, T\in {\mathscr {T}}_H\}$ for the ansatz space $V_{H}^{\ell }$ that is associated with a basis $\{g_{T,\ell ,\varepsilon }^\textrm{loc}\,|\, T\in {\mathscr {T}}_H\}$ of ${\mathbb {P}}^0({\mathscr {T}}_H)$ via  (4.1). The Riesz stability of the basis in the sense of Assumption 2 must be respected. However, there is still no a priori guarantee that this will hold, but we believe that the approach below helps to get a Riesz basis, which may be checked a posteriori.

For simplicity, we take $\varOmega$ as the unit hypercube in *d* dimensions, i.e., $\varOmega =(0,1)^d$, discretized by means of a quadrilateral mesh ${\mathscr {T}}_H$. Given $\ell \ge 1$, we choose an element $T\in {\mathscr {T}}_H$ and consider the corresponding patch $\omega = N^\ell (T)$. In a first step, for each element $K\in {\mathscr {T}}_{H,\omega }$ in the patch mesh, we compute the response of the solution operator restricted to the patch, denoted by ${\mathscr {A}}_{\omega }^{-1}$, to its characteristic function ${\textbf{1}}_{K}$, i.e., ${\mathscr {A}}_{\omega }^{-1}{\textbf{1}}_{K}$. By construction, the target basis function $\varphi _{T,\ell ,\varepsilon }^\textrm{loc}$ is in the span of these $\#{\mathscr {T}}_{H,\omega }\approx \ell ^d$ local responses. In a second step, we search for the function $\varphi ^\textrm{loc}_{T,\ell ,\varepsilon } \in {\text {span}}\left\{ {\mathscr {A}}_{\omega }^{-1}{\textbf{1}}_{K} | K\in {\mathscr {T}}_{H,\omega } \right\}$ in this low-dimensional space by minimizing normal derivatives subject to a unit mass constraint as presented in (4.2). The corresponding eigenvector $(c_K)_{K\in {\mathscr {T}}_{H,\omega }}$ contains the coefficients of the expansion of $\varphi _{T,\ell ,\varepsilon }^\textrm{loc}$ in terms of the local responses. At the same time, the coefficients are the values of $g_{T,\ell ,\varepsilon }^\textrm{loc}$ in the elements of the patch.

Unfortunately, the smallest eigenvalue may not be simple, or there might be a cluster of small eigenvalues. Then a particular choice of eigenfunction may not always be favourable with regard to the global stability of the basis in the sense of Assumption 2. Especially for patches that touch the boundary of the global domain $\varOmega$ [40, Appendix B] and in the regime of high convection, an additional optimization step ensures linear independence of the functions computed in different patches. For this purpose, we incorporate eigenfunctions associated with a certain range of the lowermost eigenvalues. Given all eigenvalues $\lambda _{1}\le \lambda _2\le \dots \le \lambda _{\#{\mathscr {T}}_{H,\omega }}$ and some parameter $p\ge 1$, we choose all indices $1\le i \le \#{\mathscr {T}}_{H,\omega }$ so that

6.1 $$\begin{aligned} \frac{\lambda _{i}}{\lambda _{\#{\mathscr {T}}_{H,\omega }}} \le \max \left\{ \left( \frac{\lambda _{1}}{\lambda _{\#{\mathscr {T}}_{H,\omega }}}\right) ^{\frac{1}{p}} , 1e{-}10 \right\} , \end{aligned}$$

and we denote the resulting set of indices by *I*. The choice $p = 1$ reflects the case where only the smallest (potentially multiple) eigenvalue is used, and thus we use $p>1$ in our implementation.

Among these candidate functions with close to minimal normal derivative at the boundary of the patch, we choose the one that maximizes a weighted $L^2(\omega )$-norm under the unit mass constraint. The piecewise constant weight function is zero in the central element *T* and grows in a *b*-dependent way with a certain distance from the central element. Let us introduce the midpoints $m_{T},m_{K}\in {\mathbb {R}}^d$ of the central element and an element of the patch, respectively. We define the relation between elements as

$$\begin{aligned} {\text {rel}}(T,K) {:}{=} H^{-1} (m_{K} - m_{T}) \in {\mathbb {Z}}^{d}, \end{aligned}$$

and introduce for each element $K\in {\mathscr {T}}_{H,\omega }\setminus T$ its weight by

6.2 $$\begin{aligned} w_{K} {:}{=} \left\| {\text {rel}}(T,K) - \frac{b(m_{T})}{\left\| b(m_{T})\right\| _{2}}\right\| _{\ell ^\infty }^{p_{w}}. \end{aligned}$$

**Fig. 4:**
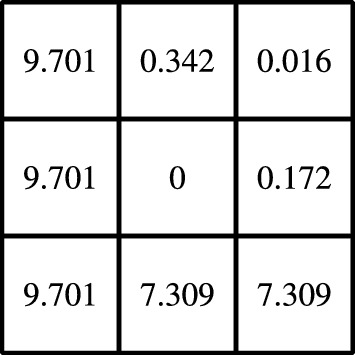
Weights $w_{K}$ for an element that does not reach the boundary, for constant velocity *b* as given in (7.1), and $p_{w} = 2$

Here $p_{w}\ge 1$ is a parameter that needs to be chosen. With (6.2) we ensure that the elements in the *b* direction are penalized less, which accounts for the natural shift of the basis due to convection. For a realization of an order 1 patch, see Fig. 4.

Eventually, we search the function in the space of the previously selected candidate right-hand sides ${\text {span}}\left\{ g_{T,\ell ,\varepsilon ,i} | i\in I\right\}$ that minimizes a weighted $L^2(\omega )$-norm subject to the unit mass constraint. This constraint minimization is realized by computing the smallest eigenvalue of the symmetric positive definite matrix

6.3 $$\begin{aligned} \left( \frac{1}{\left\| g_{T,\ell ,\varepsilon ,i}\right\| _{L^2(\omega )}\left\| g_{T,\ell ,\varepsilon ,j}\right\| _{L^2(\omega )}}\sum _{K\in {\mathscr {T}}_{H,\omega }} \int _{K} w_{K} g_{T,\ell ,\varepsilon ,i}g_{T,\ell ,\varepsilon ,j} \, \textrm{d}x\right) _{i,j\in I}. \end{aligned}$$

In this way, we compute for every element *T* of the coarse mesh ${\mathscr {T}}_H$ the basis function $\varphi ^\textrm{loc}_{T,\ell ,\varepsilon }$ and hence build the space $V_{H,\ell }$. From our numerical experiments, the choices $p = 3$ and $p_{w}=2$ produce good results. In Algorithm 1 we detail the full algorithm for the computation of the super-localized basis.

**Algorithm 1:**
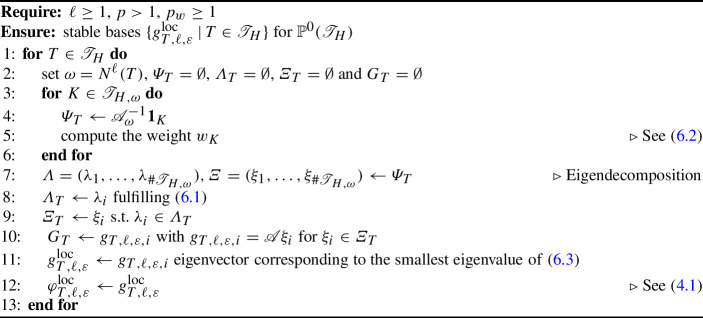
Basis selection

Although our approach gives good results in the numerical experiments in Sect. 7, we believe that it can be improved. In particular, the choice of the two-step optimization leaves room for improvement to ensure favorable locality and stability at the same time. Nevertheless, this is the best optimization we have found so far. Another way to achieve effective stabilization may be to use partiton of unity techniques, as suggested in [35].

## Numerical experiments

In this section, we demonstrate the performance of our method. For this purpose, we briefly introduce the general configuration. All our experiments have been performed in Matlab. The computational domain $\varOmega$ is given as a unit hypercube in *d* dimensions, i.e., $\varOmega =(0,1)^d$. We introduce a fine quadrilateral mesh ${\mathscr {T}}_h$, which resolves the small parameter $\varepsilon$ and is used to compute reference solutions and localized basis functions using the standard Galerkin FE method on the space of piecewise bilinear polynomials. In addition, we consider a coarse quadrilateral mesh ${\mathscr {T}}_H$ as the target scale, which does not resolve $\varepsilon$. We emphasize that on this mesh, the degrees of freedom of the FEM correspond to the vertices of the mesh, while the degrees of freedom of the SLOD correspond to the elements. Thus, in principle, the SLOD has fewer degrees of freedom, but an increasing localization parameter results in a slightly denser matrix.

### Two-dimensional experiment

We start by presenting two-dimensional experiments and compare our approach with the streamline upwind/Petrov–Galerkin (SUPG) method (see [34]) that we briefly recall below. Let $U_H$ denote the standard Galerkin FE space of piecewise bilinear polynomials on the coarse mesh ${\mathscr {T}}_H$. The SUPG approximation $u^{{\text {SUPG}}}_H\in U_H$ satisfies, for all $v_H\in U_H$

$$\begin{aligned} B_{{\text {SUPG}}}(u_H^{{\text {SUPG}}},v_H) = F_{{\text {SUPG}}} (v_H), \end{aligned}$$

with

$$\begin{aligned} B_{{\text {SUPG}}}(u_H^{{\text {SUPG}}},v_H){:}{=} a(u_H^{{\text {SUPG}}},v_H) + \delta _{{\text {SUPG}}}\sum _{T\in {\mathscr {T}}_H}\left( b\cdot \nabla u_H^{{\text {SUPG}}},b\cdot \nabla v_H\right) _{L^2(T)} \end{aligned}$$

and

$$\begin{aligned} F_{{\text {SUPG}}} (v_H) = \langle f,v_H\rangle _{H^{-1}(\varOmega )\times H^{1}_0(\varOmega )} + \delta _{{\text {SUPG}}}\sum _{T\in {\mathscr {T}}_H}\left( f,b\cdot \nabla v_H\right) _{L^2(T)}. \end{aligned}$$

The symbol $\delta _{{\text {SUPG}}}$ denotes the stabilization parameter, and is chosen as $\delta _{{\text {SUPG}}} = \frac{H}{2 \left\| b\right\| _{2}}$.

#### Constant velocity

First, we follow the experiment from [50, Section 6]. We choose the right-hand side $f\equiv 1$ and the constant velocity field *b* as

7.1 $$\begin{aligned} b(x) = \begin{pmatrix} \cos (0.7)&\sin (0.7) \end{pmatrix}^\top . \end{aligned}$$

The singular perturbation parameter $\varepsilon$ is chosen to be $2^{-8}$. In this configuration, we expect boundary layers at the right and top boundaries. Moreover, in this situation the right-hand side is piecewise constant and hence, the first expression in our error estimate in (5.6) vanishes. Therefore, we observe the localization error.

**Fig. 5:**
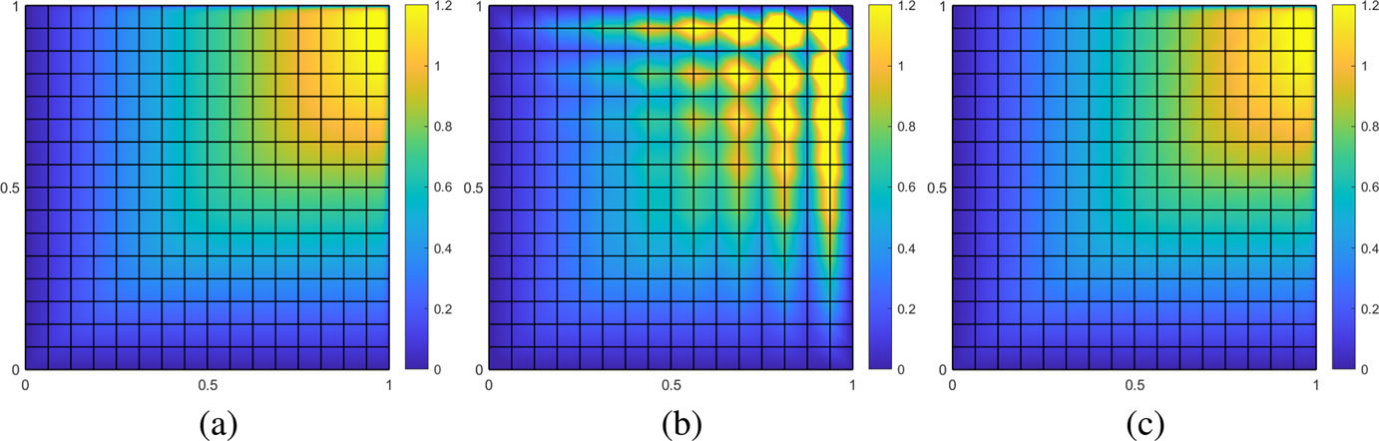
Reference solution computed on fine mesh with $h=2^{-10}$ (**a**), FE approximation (**b**) and SLOD approximation with $\ell = 1$ (**c**) on coarse mesh with $H=2^{-4}$ for the constant velocity field *b* given in (7.1), right-hand side $f\equiv 1$ and $\varepsilon = 2^{-8}$

The mesh size *h* of the fine mesh ${\mathscr {T}}_h$ is chosen as $h = 2^{-10}$. Figure 5 shows the corresponding reference solution, its FE and SLOD approximation on a coarse mesh with $H = 2^{-4}$. The localization parameter $\ell$ is chosen equal to 1.

**Fig. 6:**
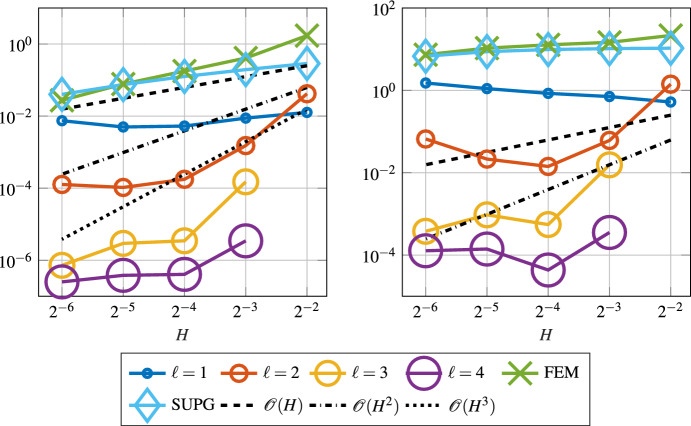
Error in $L^2$- (left) and $\left| \bullet \right| _{V}$-norm (right) for the constant velocity field *b* as in (7.1) and right-hand side $f\equiv 1$ with $\varepsilon = 2^{-8}$

We observe that the SLOD resolves the layer, whereas the classic FE approximation suffers from severe instabilities. Figure 6 shows the convergence rates of the SLOD method for different localization parameters $\ell$ and coarse mesh sizes *H* as well as the error of the FE and SUPG methods.

**Fig. 7:**
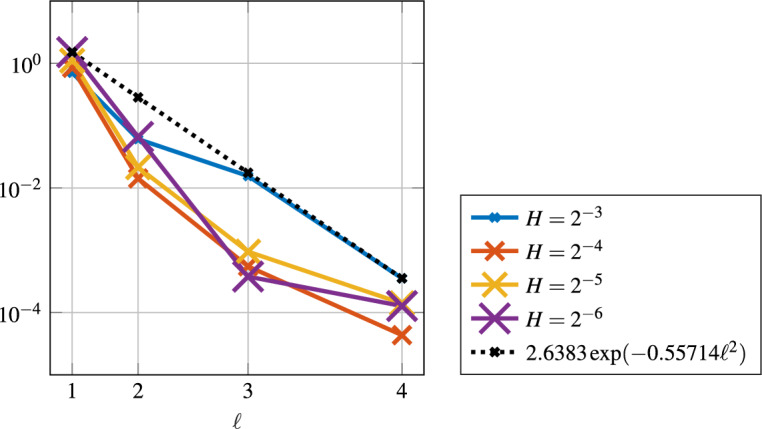
Decay of the localization error in $\left| \bullet \right| _{V}$-norm versus the localization parameter $\ell$ for $\varepsilon = 2^{-8}$ and various values of *H*

The super-exponential convergence of (4.4) is numerically verified in Fig. 7. Unfortunately, our method shows inaccuracies for refinements in *H*. Most likely, these are due to the selection of the basis functions as discussed in Sect. 6 and hence an improvement in this selection process could lead to a more accurate method. However, we chose to ensure stability and possibly lose some accuracy in return.

#### Variable velocity and non-constant right-hand side

In this example, we consider a varying velocity field *b*, given as

7.2 $$\begin{aligned} b(x) = \begin{pmatrix} 2&\,&1 + \tfrac{4}{5} \sin (4 \pi (x_{1}+1)) \end{pmatrix}^\top ,\quad \text {for all }x\in \varOmega . \end{aligned}$$

**Fig. 8:**
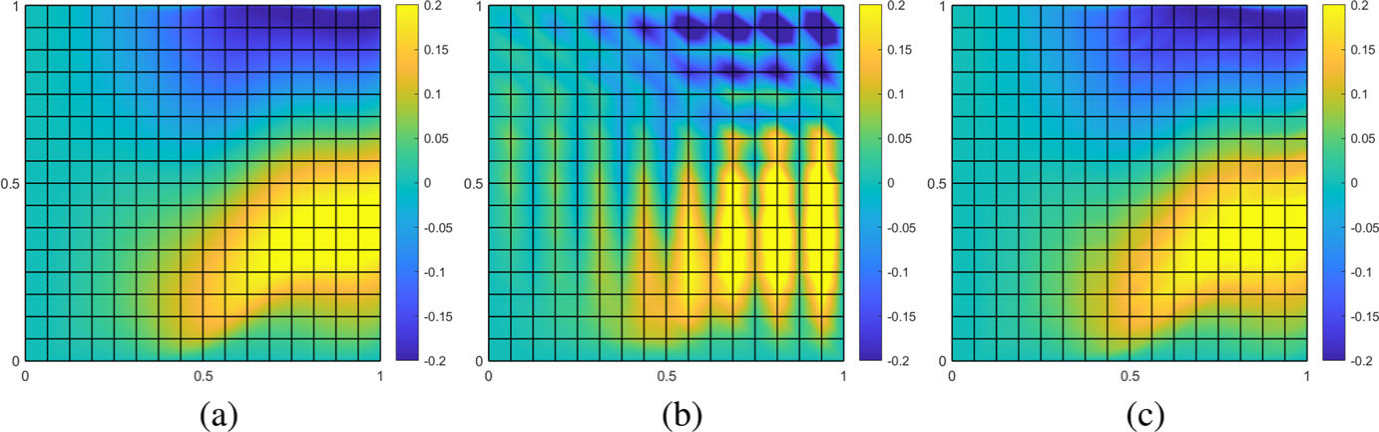
Reference solution computed on fine mesh with $h=2^{-10}$ (**a**), FE approximation (**b**) and SLOD approximation with $\ell = 1$ (**c**) on coarse mesh with $H=2^{-4}$ for the variable velocity field *b* given in (7.2), right-hand side $f= \sin (\pi x_{1}) \cos (\pi x_{2})$ and $\varepsilon = 2^{-8}$

It should be emphasized that the velocity field is divergence-free but not curl-free. Nevertheless, the numerical experiments show that the proposed multi-scale method can be satisfactorily applied beyond the assumptions of the theory. Since *b* varies in space, the penalization introduced in Sect. 6 varies with the different macroscopic cells in the coarse mesh. The flow in this example yields a boundary layer at the right boundary. We choose the non-constant right-hand side as $f(x) = \sin (\pi x_{1}) \cos (\pi x_{2})$. Thus, with respect to the error estimate in Theorem 5.1 the first expression in (5.6) does not vanish, and we expect a convergence of order one in the $\left| \bullet \right| _{V}$-norm as the right-hand side is regular enough. Figure 8 shows the reference solution as well as its FE and SLOD approximations. We again observe that the SLOD resolves the boundary layer, whereas the FEM delivers poor results.

For non-constant right-hand sides, we expect improved approximation properties of the SLOD method (5.1) with $\Pi _H f$ replaced by *f*. We will refer to such a method as SLOD-Galerkin. Note that the two methods produce different approximations and require different computational efforts. More specifically, since both methods search for an approximation in the same ansatz space ${\mathscr {V}}_H^\ell$, they share the offline phase, namely the computation of the set of operator-adapted local basis functions. On the other hand, they differ in the online phase, where the actual approximation to the solution is computed. In particular, the SLOD method is more efficient online than the SLOD-Galerkin method.

**Fig. 9:**
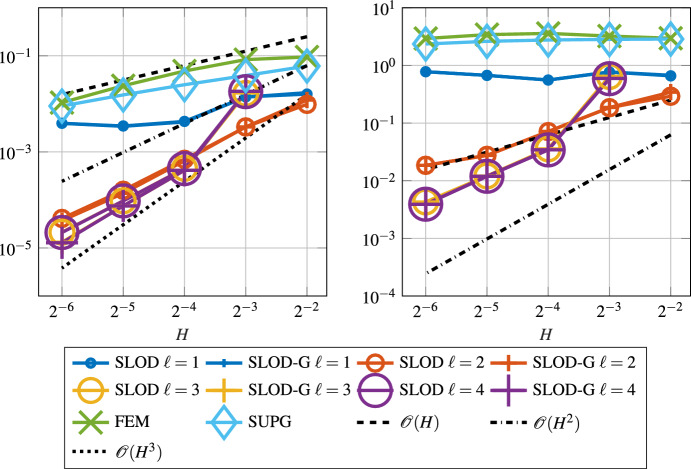
Error in $L^2(\varOmega )$- (left) and $\left| \bullet \right| _{V}$-norm (right) for the variable velocity field *b* as in (7.2) and right-hand side $f= \sin (\pi x_{1}) \cos (\pi x_{2})$. We consider the parameter $\varepsilon = 2^{-8}$ and the proposed SLOD method as well as the SLOD-Galerkin (SLOD-G)

The convergence in the $L^2(\varOmega )$ and $\left| \bullet \right| _{V}$ norms for both the SLOD and the SLOD-Galerkin is shown in Fig. 9, where we observe second order convergence in the $\left| \bullet \right| _{V}$ norm in *H* for both methods. In the $L^2(\varOmega )$ norm, the SLOD-Galerkin has a third order convergence, one order better than our proposed method, due to the extra effort to integrate the right-hand side more accurately.

### Three-dimensional experiment

As mentioned before, the variational multi-scale stabilization method from [50] only works in one or two dimensions. Here we show that the super-localized variant is capable to approximate the solution even in a three-dimensional setup. In this configuration we again choose a constant velocity field *b*, which is given as

7.3 $$\begin{aligned} b = \frac{\pi }{4}\begin{pmatrix} 1&1&1 \end{pmatrix}^\top . \end{aligned}$$

**Fig. 10:**
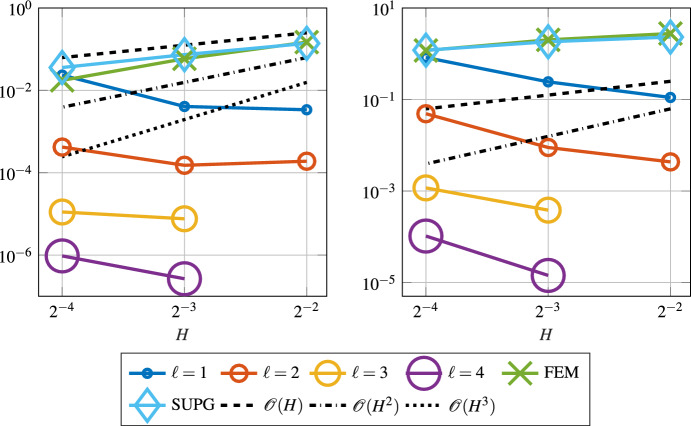
Error in the $L^2(\varOmega )$- (left) and $\left| \bullet \right| _{V}$-norm (right) for the constant velocity field *b* as in (7.3), right-hand side $f\equiv 1$ and $\varepsilon = 2^{-5}$, in the three-dimensional case

The constant right-hand side is $f\equiv 1$. We expect a boundary layer around the top right corner at $$\begin{pmatrix} 1&1&1 \end{pmatrix}^{\top }$$. In the three-dimensional setting we compute the reference solution on a mesh with $h = 2^{-6}$, which resolves the chosen $\varepsilon = 2^{-5}$. Figure 10 shows the $L^2(\varOmega )$- and $\left| \bullet \right| _{V}$-norm errors obtained for the SLOD method. As in our first experiment, due to the constant right-hand side we observe the localization error.

**Fig. 11:**
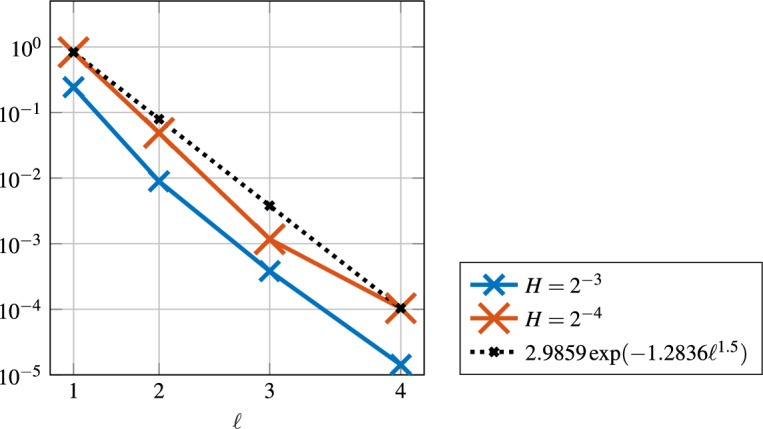
Super-exponential decay of the $\left| \bullet \right| _{V}$-norm in localization parameter $\ell$ for the three-dimensional experiment

From (4.4), for $d=3$, we deduce that the localization error behaves like $\exp (-C \ell ^{1.5})$. Figure 11 illustrates this super-exponential decay in the $\left| \bullet \right| _{V}$-norm.

## Concluding remarks and future developments

We have presented a novel multi-scale method for convection-dominated problems. The method follows the LOD framework and employs a novel super-localization strategy. The resulting SLOD significantly improves previous attempts to tackle convection-dominated problems in the under-resolved regime of large mesh Péclet numbers. While previously the SLOD largely improved the performance of already very efficient methods for model diffusion and Helmholtz problems [36, 40], the present paper demonstrates the true potential of the super-localization idea to enlarge the class of problems tractable by multi-scale methods. The numerically observed $\varepsilon$-independent convergence in two as well as three-dimensional experiments is to some extent justified by numerical analysis involving a priori and a posteriori techniques.

Among the many promising future research directions are parameterized elliptic multi-scale problems to be treated by combining SLOD with model order reduction techniques, following the ideas in [1]. It has been shown in [9] that this is conceptually possible for problems involving convection. An interesting and relevant application from the physical point of view are wave propagation and scattering problems in highly heterogeneous structures. For such an objective, the SLOD has been recently proposed in [36], and model order reduction techniques for the parametric-in-frequency problem have recently been presented (see, e.g., [13–17]). Moreover, the possible improvement of numerical stochastic homogenization methods [32, 33, 37, 39] and uncertainty quantification techniques [8, 10–12] will be analyzed.
